# Impact of microbial Aetiology on mortality in severe community-acquired pneumonia

**DOI:** 10.1186/s12879-018-3366-4

**Published:** 2018-09-04

**Authors:** Jessica Quah, Boran Jiang, Poh Choo Tan, Chuin Siau, Thean Yen Tan

**Affiliations:** 1 Department of Respiratory and Critical Care Medicine, Changi General Hospital, SingHealth, 2 Simei Street 3, Postal Code, Singapore, 529889 Singapore; 2 Department of Laboratory Medicine, Changi General Hospital, SingHealth, Singapore, Singapore; 3 Department of Advanced Nursing Practice, Changi General Hospital, SingHealth, Singapore, Singapore

**Keywords:** Virus PCR, ICU, Acute respiratory failure, Pneumonia mortality, Mixed respiratory infections, Mechanical ventilation

## Abstract

**Background:**

The impact of different classes of microbial pathogens on mortality in severe community-acquired pneumonia is not well elucidated. Previous studies have shown significant variation in the incidence of viral, bacterial and mixed infections, with conflicting risk associations for mortality. We aimed to determine the risk association of microbial aetiologies with hospital mortality in severe CAP, utilising a diagnostic strategy incorporating molecular testing. Our primary hypothesis was that respiratory viruses were important causative pathogens in severe CAP and was associated with increased mortality when present with bacterial pathogens in mixed viral-bacterial co-infections.

**Methods:**

A retrospective cohort study from January 2014 to July 2015 was conducted in a tertiary hospital medical intensive care unit in eastern Singapore, which has a tropical climate. All patients diagnosed with severe community-acquired pneumonia were included.

**Results:**

A total of 117 patients were in the study. Microbial pathogens were identified in 84 (71.8%) patients. Mixed viral-bacterial co-infections occurred in 18 (15.4%) of patients. Isolated viral infections were present in 32 patients (27.4%); isolated bacterial infections were detected in 34 patients (29.1%). Hospital mortality occurred in 16 (13.7%) patients. The most common bacteria isolated was *Streptococcus pneumoniae* and the most common virus isolated was *Influenza A*. Univariate and multivariate logistic regression showed that serum procalcitonin, APACHE II severity score and mixed viral-bacterial infection were associated with increased risk of hospital mortality. Mixed viral-bacterial co-infections were associated with an adjusted odds ratio of 13.99 (95% CI 1.30–151.05, *p* = 0.03) for hospital mortality.

**Conclusions:**

Respiratory viruses are common organisms isolated in severe community-acquired pneumonia. Mixed viral-bacterial infections may be associated with an increased risk of mortality.

## Background

The microbial aetiology of severe community-acquired pneumonia (CAP) remains varied throughout the world, with influences due to seasonal climate change, outreach of vaccination programmes and pathogen endemicity [1, 2]. Two decades ago, it was thought that viruses played a minor role in the pathogenesis of severe CAP, notwithstanding influenza epidemics [3]. Recent literature contradicts this and suggests that viruses are frequently found in severe CAP [4, 5].

Advances in molecular techniques have improved the sensitivity, accuracy and turnaround time of microbial diagnostic tests [6, 7]. The availability of highly multiplexed commercial tests kits for common viral and bacterial pathogens has enabled these tests to be performed in large numbers of patients simultaneously, and across a variety of clinical settings [8, 9]. Multiple respiratory viruses may be present concurrently, or co-exist with bacterial pathogens to cause disease [10–12]. The reported incidence of viruses in severe CAP resulting in respiratory failure ranges 7.7% to 57% [4, 5]. This variation in the detection rate of viruses reflects potential limited availability of test assays and heterogeneity of physician practices in viral microbial diagnostic tests performed [13, 14]. Postulated prohibitive factors against the routine performance of viral diagnostics tests in patients with severe CAP may include a lack of clear clinical guidelines, perceived low cost-effectiveness and the paucity of effective anti-viral therapies for respiratory viruses other than influenza.

The primary aim of our study was to determine the risk association of microbial aetiologies with hospital mortality in severe CAP, utilising a diagnostic strategy that incorporated molecular testing. Our primary hypothesis was that respiratory viruses were important causative pathogens in severe CAP and was associated with increased mortality when present with bacterial pathogens in mixed viral-bacterial co-infections.

## Methods

### Study design

This was a retrospective cohort study performed in an 18-bed medical intensive care unit of a 1060-bed tertiary teaching hospital, in Singapore. Ethics approval was obtained from the SingHealth Centralised Institutional Review Board (CIRB 2013/685/FP), with waiver of consent.

### Inclusion criteria

Adults above the age of 21 admitted to the medical intensive care unit with severe CAP from January 2014 to July 2015 were included. Written and electronic medical records were reviewed. CAP was defined as an acute infection of the lung parenchyma associated with acute chest radiographic infiltrates and 2 or more of the following: fever of 38 °C or higher or hypothermia of 36 °C or less; a new cough; dyspnea not explained by other reasons; worsening cough or change in respiratory secretions in a patient with pre-existing chronic cough. These symptoms should have been present at the time of, or within 48 h of, hospital admission. Chest radiographs reported by the hospital radiologists were obtained to confirm the presence of radiographic pulmonary infiltrates.

Patients with shock requiring vasopressor support, mechanically ventilated or who have 3 out of 9 minor criteria for pneumonia severity as defined by the Infectious Diseases Society of America/American Thoracic Society, are considered to have severe CAP [15]. The 9 minor criteria include: respiratory rate of 30 breaths per minute or greater; PaO_2_/FiO_2_ ratio equal or less than 250; multi-lobar pulmonary infiltrates; confusion or disorientation; blood urea nitrogen equal or greater than 20 mg/dL; leukopenia with white blood cell count of less than 100,000 cells/mm^3^; hypothermia indicated by core body temperature less than 36 °C; hypotension requiring aggressive fluid resuscitation.

### Exclusion criteria

All patients with prior hospitalisation within 30 days of study enrolment; on active chemotherapy for neoplastic diseases; receiving renal replacement therapy by haemodialysis and prisoners were excluded. Immunocompromised patients were not excluded as they were likely to have CAP microbial pathogens as well as opportunistic infections.

### Collection of microbiological samples

The time of admission to the intensive care unit was verified with electronic records. All patients had at least 1 set of aerobic and anaerobic blood cultures performed within 24 h of admission. Routine collection of endotracheal aspirates for Gram stain and semi-quantitative bacterial cultures occurred within 24 h of intubation. Viral and atypical pathogen polymerase chain reaction (PCR) amplification tests were collected from endotracheal aspirates. Sputum samples were sent for bacterial aerobic cultures, while nasopharyngeal swabs were performed for atypical pathogen and viral PCR amplification tests for subjects who did not require intubation. Urinary samples were tested for the presence of urinary *Streptococcus pneumoniae* antigen and *Legionella pneumophilia* serogroup 1 antigen in patients without anuria. Where indicated, acid-fast staining and mycobacterial cultures of respiratory samples were performed.

### Laboratory testing for viruses, atypical organisms, bacteria and melioidosis

Nucleic acid was extracted from swabs or respiratory samples using EZ1 Virus mini kit (Cat No. 955134, Qiagen, Germany) performed on a semi-automated system (EZ1, Qiagen, Germany), and stored at -70 °C for more detailed molecular testing. Besides routine standard clinical testing, additional multiplex real-time PCR testing was performed for each extracted sample.

Anyplex RV16 PCR (Cat No. RV7G01Y, Seegene, Korea) was performed for detection *of Influenza A virus, Influenza B virus, Human respiratory syncytial virus A, Human respiratory syncytial virus B, Human adenovirus, Human metapneumovirus, Human coronavirus 229E/NL63/OC43, Human parainfluenza virus 1/2/3/4, Human rhinovirus A/B/C, Human enterovirus and Human bocavirus 1/2/3/4*.

Anyplex RB5 PCR (Cat No. RB7500Y, Seegene, Korea) was performed for detection of *Chlamydophila pneumoniae*, *Mycoplasma pneumoniae*, *Legionella pneumophila*, *Bordetella pertussis*, and *Bordetella parapertussis*. Additional multiplex assays were performed for the detection of *Legionella species* (non-*pneumophila*), *Chlamydia psittaci*, and *Burkholderia pseudomallei*. Finally, bacterial identification was performed with Vitek MS (Maldi-tof) supplemented by conventional biochemical and identification methods when indicated.

### Clinical data collection

Written and electronic medical records were reviewed for clinical data and laboratory indices. The most severe value was recorded for analysis if any blood test was repeated more than once within 48 h after intensive care admission. Serum C-reactive protein was measured by particle enhanced immunoturbidimetry (Cobas® CRPL3) and serum procalcitonin was measured using sandwich immunoassay (Cobas® Elecsys BRAHMS PCT).

Variables such as the Acute Physiology and Chronic Health Evaluation II (APACHE II) severity score, presence of shock, mechanical ventilation, intensive care unit length of stay, and mortality were captured prospectively as part of administrative clinical care audits. Empirical antibiotics were deemed to be adequate if it adhered to the hospital antimicrobial guidelines. Combination therapy with beta-lactams and macrolides were recommended. Where melioidosis was suspected, carbapenems were preferred to other classes of beta-lactams.

### Statistical analysis and outcome measures

Categorical variables were expressed as number (percentages) and normally distributed quantitative variables were expressed as mean (± standard deviation). Categorical variables were compared with Chi-square test or Fisher’s exact test, and quantitative variables were compared with T-test or Mann-U Whitney test.

The primary outcome measure was all-cause hospital mortality. Demographic and disease variables were included in the univariate and multivariable logistic regression model. Pneumonia symptoms were excluded from regression analysis as there were no known associations with mortality in severe CAP. The variable for number of co-existent pathogens was excluded from regression analysis due to significant overlap with mixed viral-bacterial co-infections, which was included in the multivariate model. The following severity indicators: shock, altered mentation and ventilator support were not included as these variables are incorporated in the APACHE II severity score. *Mycobacterium tuberculosis* was classified as a bacterium for the logistic regression. Demographic and disease variables were then compared between patients with and without virus infections, for identification of potential risk factors associated with acquisition of respiratory viral infections. A *p*-value of < 0.05 was considered significant. Missing data was excluded from univariate and multivariate analysis. STATA Special Edition version 15.0 (Statacorp LLC, Texas, USA) was used for statistical analysis.

## Results

One hundred seventeen patients were admitted to the medical intensive care unit for severe CAP within the study period. Baseline characteristics of patients who suffered in-hospital mortality (*n* = 16, 13.7%) were compared with patients who survived, in Table 1. Of note, the patients who did not survived were older compared to survivors (68.5 years vs 61.6 years, *p* = 0.11). Other baseline demographic and co-morbidity variables were not significantly different.

**Table 1:** Patient characteristics in relation to hospital mortality

Characteristics	All patients (*n* = 117)	Survivors (*n* = 101)	Non-survivors (*n* = 16)	*p*-value
Demographics				
Age, years	62.5 ± 16.1	61.6 ± 16.5	68.5 ± 11.7	0.11
Male	73 (62.4)	62 (61.4)	11 (68.8)	0.78
Comorbid illness				
Diabetes Mellitus	36 (30.8)	31 (30.7)	5 (31.3)	0.96
Hypertension	67 (57.3)	58 (57.4)	9 (56.3)	0.93
Cardiac disease	26 (22.2)	20 (19.8)	6 (37.5)	0.11
Chronic lung disease	23 (19.7)	22 (21.8)	1 (6.3)	0.19
Cerebrovascular disease	14 (12.0)	11 (10.9)	3 (18.8)	0.41
Chronic liver disease	1 (0.9)	0 (0)	1 (0.06)	0.14
End-stage renal failure	5 (4.3)	3 (3.0)	2 (12.5)	0.14
Symptoms				
Fever	71 (60.7)	63 (62.4)	8 (50)	0.35
Cough	97 (82.9)	84 (83.2)	13(81.3)	0.85
Purulent Sputum	63 (53.9)	57 (56.5)	6 (37.5)	0.16
Dypsnea	82 (70.1)	70 (69.3)	12 (75.0)	0.65
Radiological features				
Localised lung infiltrates	35 (29.9)	30 (29.7)	5 (31.2)	0.90
Multi-lobar lung infiltrates	82 (70.1)	71 (70.3)	11 (68.8)	0.90
Pleural effusion	15 (12.8)	12 (11.9)	3 (18.8)	0.43
Laboratory Indices				
Serum C- reactive protein^a^ (mg/L)	157.2 ± 114.8	157.9 ± 118.3	152.9 ± 93.9	0.87
Serum procalcitonin^b^ (ug/L)	19.8 ± 34.6	17.0 ± 32.3	38.4 ± 44.5	0.025
Microbiological factors				
Number of co-existent pathogens				
-0	33 (28.2)	30 (29.7)	3 (18.8)	0.010
-1	64 (54.7)	58 (57.4)	6 (37.5)	
-2 or more	20 (17.1)	13 (12.9)	7 (43.8)	
Microbial aetiologies				
- No identified pathogen	33 (28.2)	30 (29.7)	3 (18.8)	0.008
- Only bacterial pathogens present	27 (23.1)	26 (25.7)	1 (6.3)	
- Only viral pathogens present	32 (27.4)	27 (26.7)	5 (31.3)	
- Mixed viral-bacterial infection	18 (15.4)	11 (10.9)	7 (43.8)	
- Atypical pathogens	7 (6.0)	7 (6.9)	0 (0)	
Appropriate empirical antibiotics	108 (92.3)	94 (93.1)	14 (87.5)	0.44
Severity indicators				
APACHE II	22.1 ± 9.4	20.8 ± 8.6	30.1 ± 10.3	< 0.001
Shock	69 (56.0)	54 (53.5)	15 (93.8)	0.002
Altered mentation	22 (18.8)	16 (15.8)	6 (37.5)	0.039
Ventilatory support				
-Invasive	108 (92.3)	92 (91.1)	16 (100.0)	0.46
-Non-invasive	3 (2.6)	3 (3.0)	0 (0)	
-Supplemental oxygen	6 (5.1)	6 (5.9)	0 (0)	

Data are presented as number (%), mean ± standard deviation. APACHE = Acute Physiology and Chronic Health Evaluation. ^a^ Data was missing for 4 patients. ^b^Data was missing for 1 patient

Blood cultures, respiratory specimen cultures and respiratory specimen PCR testing for viruses and atypical bacteria were performed in all patients. Results of microbiological tests are presented in Table 2. Causative microbial aetiologies were identified in 71.8% of patients with specific organisms presented in Table 3. Majority of pathogens identified were respiratory viruses and bacteria. Viruses were found in 42.7% of patients (*n* = 50) with the most common virus being *Influenza A.* 35.7% (*n* = 10) of patients with *Influenza A* received empirical oseltamivir on the basis on clinical suspicion. Bacterial infections were found in 38.5% of patients (*n* = 45). 15.4% of patients (*n* = 18) had mixed virus and bacterial co-infections. 32 patients (27.4%) had only viral pathogens and 27 patients (23.1%) had only bacterial pathogens, found as the microbial aetiology for severe CAP.

**Table 2:** Microbiological investigations performed and its yield

Microbiological Test	Performed (*n* = 117)	Yield of test
Urine Streptococcal Antigen	111 (94.9)	18 (16.2)
Urine Legionella Antigen	111 (93.2)	2 (1.8)
Respiratory cultures	117 (100)	30 (25.6)
Acid-Fast Bacilli Smear	109 (93.2)	2 (1.8)
Mycobacterium culture	51 (43.6)	3 (5.9)
Blood cultures	117 (100)	14 (12.0)
Atypical bacterial and viruses Multiplex PCR	117 (100)	57 (48.7)

Data are presented as number (%)

Yield of test is present as number (% positive samples over total samples performed)

**Table 3:** Distribution of microbial pathogens detected in study cohort

Microbial Pathogens	Study Cohort, *n* = 117 (%)
Viruses	
Influenza A*	28 (24.0)
*Influenza B*	5 (4.3)
*Rhinovirus*	6 (5.1)
*Human Metapneumovirus*	5 (4.3)
*Adenovirus*	3 (2.6)
*Coronavirus*	2 (1.7)
*Respiratory Syncytial Virus*	2 (1.7)
*Parainfluenza*	1 (0.9)
Bacteria	
*Streptococcus pneumoniae*	19 (16.3)
*Staphylococcus aureus*	6 (5.1)
*Klebsiella pneumoniae*	5 (4.3)
*Pseudomonas aeruginosa*	4 (3.4)
*Haemophilus influenzae*	3 (2.6)
*Moraxella cataharrlis*	2 (1.7)
*Achromobacter*	1 (0.9)
*Escherichia coli*	1 (0.9)
*Burkholderia pseudomallei*	1 (0.9)
*Nocardia*	1 (0.9)
Atypical organisms	
*Mycoplasma pneumoniae*	4 (3.4)
*Legionella*	3 (2.6)
*Mycobacterium tuberculosis*	3 (2.6)

Data are presented as number (%). *9 out of 28 *Influenza A* samples were serotype H1N1

Using hospital mortality as the primary outcome, univariate logistic regression was performed (Table 4). Patients who did not survive had a higher APACHE II score and higher serum procalcitonin levels. The microbiological aetiology that was significantly associated with increased hospital mortality was the detection of mixed viral-bacterial co-infections.

**Table 4:** Univariate and multivariate logistic regression for hospital mortality

Variable	Univariate analysis		Multivariate analysis	
	O.R. (95% CI)	*p*-value	Adjusted O.R. (95% CI)	*p*-value
Age	1.03 (0.99, 1.07)	0.114	1.01 (0.94, 1.09)	0.738
Male	0.72 (0.23, 2.24)	0.573	0.37 (0.05, 2.61)	0.320
Diabetes Mellitus	1.03 (0.33, 3.20)	0.964	1.32 (0.13, 13.04)	0.814
Hypertension	0.95 (0.33, 2.76)	0.930	0.57 (0.08, 4.18)	0.578
Cardiac disease	2.43 (0.79, 7.48)	0.122	4.85 (0.63, 37.7)	0.131
Chronic lung disease	0.24 (0.23, 1.91)	0.178	0.34 (0.03, 4.38)	0.404
Cerebrovascular disease	1.89 (0.46, 7.68)	0.375	5.59 (0.58, 53.7)	0.136
End-stage renal failure	4.67 (0.72, 30.4)	0.108	7.23 (0.18, 296.8)	0.297
Serum C-Reactive Protein	1.00 (1.00, 1.00)	0.693	1.00 (0.99, 1.01)	0.872
Serum Procalcitonin	1.01 (1.00, 1.03)	0.035	1.03 (1.00, 1.05)	0.044
Multilobar lung infiltrates	1.08 (0.34, 2.36)	0.900	2.14 (0.36, 12.51)	0.400
Pleural effusion	1.71 (0.43, 6.89)	0.449	7.97 (0.36, 178.40)	0.191
APACHE II	1.12 (1.05, 1.19)	0.001	1.15 (1.03, 1.28)	0.014
Appropriate empirical antibiotics	0.52 (0.10, 2.77)	0.444	2.59 (0.013, 522.06)	0.726
Microbial pathogens				
*-*No isolates	1			
*-*Bacteria only	0.30 (0.03, 3.07)	0.312	0.14 (0.004,2.27)	0.143
*-*Viruses only	1.85 (0.40, 8.49)	0.428	4.63 (0.47, 45.53)	0.189
*-*Mixed viral-bacterial co-infections	6.36 (1.39, 29.1)	0.017	13.99 (1.30, 151.05)	0.030
*-*Atypical infection	Omitted		Omitted	

Data are presented as odds ratio (95% confidence interval)

*APACHE* Acute Physiology and Chronic Health Evaluation

On multivariate analysis, APACHE II severity score, serum procalcitonin and mixed viral-bacterial co-infections remained significantly associated with increased adjusted odd ratios for hospital mortality. While APACHE II severity score is known to be predictive for mortality in severe CAP, the study found that the presence of mixed viral-bacterial co-infections was associated with increased hospital mortality by an adjusted odds ratio of 13.99 (95% confidence interval 1.30, 151.05, *p* = 0.030).

The patients with respiratory viruses detected (both isolated viral pathogens and mixed viral-bacterial co-infections) were compared with patients who did not have any respiratory virus infections, in Table 5. The mean serum C-reactive protein was found to be greater (178.1 ± 117.6 mg/L vs. 127.8 ± 105.0 mg/L) in patients without respiratory viruses compared to patients with detection of respiratory viruses (Table 5).

**Table 5:** Patient characteristics in relation to respiratory virus detection

Characteristics	Viruses detected (*n* = 50)	Viruses not detected (*n* = 67)	*p*-value
Demographics			
Age, years	64.3 ± 14.9	61.3 ± 17.0	0.33
Male	28 (56.0)	45 (67.2)	0.22
Comorbid illness			
Diabetes Mellitus	18 (36.0)	18 (26.9)	0.32
Hypertension	32 (64.0)	35 (52.2)	0.20
Cardiac disease	13 (25.0)	13 (19.4)	0.40
Chronic lung disease	11 (22.0)	12 (17.9)	0.58
Cerebrovascular disease	5 (10.0)	9 (13.4)	0.57
Symptoms			
Fever	31 (62.0)	40 (59.7)	0.80
Cough	43 (86.0)	54 (80.1)	0.44
Purulent Sputum	30 (60.0)	33 (49.3)	0.25
Dypsnea	35 (70.0)	47 (70.1)	0.99
Radiological features			
Multilobar lung infiltrates	36 (72.0)	46 (68.7)	0.70
Pleural effusion	5 (10.0)	10 (14.9)	0.43
Laboratory Indices			
Serum C- Reactive Protein^a^ (mg/L)	127.8 ± 105.0	178.1 ± 117.6	0.021
Serum Procalcitonin^b^ (ug/L)	16.0 ± 30.4	22.5 ± 37.4	0.32
Severity indicators			
APACHE II	22.3 ± 9.6	21.9 ± 9.3	0.82
Shock	30 (60.0)	39 (58.2)	0.85
Altered mentation	11 (22.0)	11 (16.4)	0.45
Ventilatory support			
-Invasive	47 (94.0)	61 (91.0)	0.84
-Non-invasive	1 (2.0)	3 (4.5)	
-Supplemental oxygen	2 (4.0)	4 (6.0)	

Data are presented as number (%), mean ± standard deviation

*APACHE* Acute Physiology and Chronic Health Evaluation

^a^Data was missing for 3 patients. ^b^Data was missing for 1 patient

## Discussion

Respiratory virus infection as a cause of severe acute respiratory distress syndrome is well-established in literature [16–18]. However, its significance as a contributory pathogen in the outcomes of severe CAP is uncertain. In this study, we showed that respiratory viruses were as commonly found as bacteria (42.7% vs 38.5%), as an aetiological pathogen. Mixed viral-bacterial co-infections occurred in 15.4% of patients and was associated with an adjusted odds ratio of 13.99 for hospital mortality.

The impact of respiratory viruses on the prognosis of severe CAP remains unclear with recent studies demonstrating contradictory results. Fisher et al., in a prospective 1-year microbiologic survey of nosocomial pneumonia and CAP complicated by respiratory failure, showed that 21.7% of patients had viral infections, which was associated with a hospital mortality of 36.7% [19]. However, Siow et al., found that viral infections were independently associated with lower hospital mortality compared to other microbial aetiologies, with an adjusted odds ratio of 0.12 (CI 0.2–0.99; *p* = 0.049) [20].

In light of these findings, accurate characterisation of the impact of microbial aetiology on the outcomes of severe CAP is required to influence future development of rapid molecular diagnostics assays and novel antimicrobial therapies that would target both viruses and bacteria [21, 22].

Piralla et al. reviewed the microbiological data of severe CAP in Northern Italy during winter-spring seasons over 7 years and found that 54.6% of patients had one or more respiratory viruses identified [23]. The 2 most common viruses isolated were *Influenza A* and *Rhinovirus*, similar with our findings. While our study was performed in a tropical country, local microbiological surveillance has shown that influenza epidemics occur twice annually [24]. This would mean that 3 influenza seasons occurred over the course of this study.

The molecular mechanisms in the viral pathogenesis of severe pneumonia are most well studied in *Influenza A* and *Streptococcus pneumoniae* co-infections. Viral infections alter host immune responses that increase susceptibility to bacterial infection through viral-induced interferons [25–27]. On clinical suspicion alone, only 35.7% (*n* = 10) of patients with *Influenza A* in this study received empirical oseltamivir. The authors postulate that incorporation of early *Influenza A* diagnostic tests may decrease the time to effective anti-viral therapies.

The second most common virus detected in our study was *Rhinovirus* (*n* = 6)*.* The association of *Rhinovirus* with severe pneumonia has previously been shown in a surveillance program for Middle East respiratory syndrome *Coronavirus* in Saudi Arabia [28]. Its genotypes A to C are associated with severe pneumonia, with in-hospital mortality rates from 10.7 to 15.5% [29]. Patients who are immunocompromised or who have chronic lung disease are most at risk [30].

There are several reasons why viral infections may have been less prominent as a cause of severe CAP in prior decades. Firstly, Grève et al. performed a prospective observational study on physician practices in the use of respiratory virus diagnostics demonstrating that despite clinical guideline recommendations on testing of respiratory viruses during influenza season, less than half of patients admitted to the intensive care unit with pneumonia were tested for viral pathogens [14]. This may have led to under-recognition of the true significance of viral pathogens and mixed viral-bacterial infections, on outcomes in severe pneumonia.

Other factors which may have contributed to under-diagnosis of viral pneumonias include the unavailability or cost of molecular diagnostic assays, and the lack of effective anti-viral therapies [31]. However, the authors argue that in this age of globalisation, highly virulent respiratory viruses have the potential to spread rapidly. Constant surveillance is required to detect outbreaks and for the implementation of isolation precautions in a timely manner [32, 33]. Understanding the significance of respiratory viruses in the pathogenesis of severe CAP would guide administrators with resource allocation when implementing vaccination programs for at-risk populations.

The high rates of compliance with performing respiratory aerobic cultures, blood aerobic and anaerobic cultures in this study, were in accordance with sepsis guidelines [34]. The most common bacterial pathogen found was *Streptococcus pneumoniae* (16.3%), which is consistent with the known epidemiology of CAP globally [35–37]. Gadsby et al., in a prior study, was able to demonstrate a bacterial yield of 81.1% when respiratory specimens from patients with CAP were tested with bacterial multiplex PCR [38]. Incorporating the use of bacterial multiplex PCR in future studies, may increase the rate of bacteria detection, and shed light on potential molecular synergisms between specific viruses and bacteria in the pathogenesis of severe CAP.

Pulmonary tuberculosis is endemic in the region where this study was performed. In our study, 3 patients had tuberculosis, one of whom had concomitant *Adenovirus* infection while another had *Streptococcus pneumoniae.* The initial presentation of pulmonary tuberculosis with clinical features consistent with severe CAP has been described by Tseng et al. [39], where 4% of patients with pulmonary tuberculosis presented with respiratory failure or septic shock. The authors postulate that pulmonary tuberculosis may play a role in increasing host susceptibility to severe infection with CAP organisms.

The authors recognise that there were several limitations to this study. Firstly, while first-dose antibiotics would have been administered as soon as sepsis is identified, we are unable to accurately define the time between administration of antibiotics and collection of specimens for microbiological assessment. This may affect the yield of bacterial pathogens recovered from blood and respiratory cultures despite bacterial sampling occurring within 24 h of intensive care admission. The yield of bacterial pathogens of this study, however, was similar with other prospective studies in severe CAP that have been performed and is therefore likely representative of the true bacterial epidemiology [37, 40, 41].

Secondly, the inherent retrospective nature of this study increases the risk of bias in data collection. However, as part of pre-established intensive care unit clinical audits and with the availability of national health records, clinical data such as participant demographics, co-morbid illnesses and severity indicators such as APACHE II were established at the time of intensive care admission and stored prospectively.

Thirdly, given the high population density of the country (3rd in the world) where this study was performed, the microbial epidemiology of severe CAP may only be extrapolated to urban settings. The study is a single-centre survey conducted in 1 of the 6 acute general hospitals serving a population of 5.6 million in a land area of 721.5 Km^2^. Hence, the epidemiology may lack generalisability when extrapolated to other tropical countries.

The fourth limitation is that we included 5 patients who were immunocompromised with typical CAP organisms and survived. They may potentially have had opportunistic infections that were not detected, however, as they did not contribute to the mortality outcomes, we felt that the microbiological data contributed by these patients were useful in the understanding of the prevalence of various classes of CAP organisms. Lastly, another potential limitation was that vaccination records could not be retrieved, and we were unable to ascertain its influences on microbial aetiologies of severe pneumonia in our study population.

The main strength of the study is the characterisation of the epidemiology of microbial pathogens in severe CAP. We were able to show that in a tropical environment, the viral and bacterial pathogens associated with severe CAP were similar with regions with a seasonal climate. Despite a lower-than-expected mortality rate for severe CAP in our study (13.7%) compared with international data [41–44], we were able to demonstrate that mixed viral-bacterial co-infections were independently associated with hospital mortality.

## Conclusion

Respiratory viruses are important causative pathogens in severe CAP and are associated with increased risk of mortality when present with bacterial pathogens in mixed viral-bacterial co-infections.

## Abbreviations

APACHE II: Acute Physiology and Chronic Health Evaluation II
CAP: Community-acquired pneumonia
PCR: Polymerase chain reaction
